# Demonstrating the Applicability of Proton Transfer Reaction Mass Spectrometry to Quantify Volatiles Emitted by the Mycoparasitic Fungus *Trichoderma atroviride* in Real Time: Monitoring of *Trichoderma*-Based Biopesticides

**DOI:** 10.1021/jasms.3c00456

**Published:** 2024-05-06

**Authors:** Franziska Lochmann, Daniel Flatschacher, Verena Speckbacher, Susanne Zeilinger, Valentina Heuschneider, Stephanie Bereiter, Arne Schiller, Veronika Ruzsanyi

**Affiliations:** † Institut für Atemgasanalytik, Universität Innsbruck, Innrain 52a and 80-82, A-6020 Innsbruck, Austria; ‡ Institut für Mikrobiologie, Universität Innsbruck, Technikerstrasse 25d, A-6020 Innsbruck, Austria

## Abstract

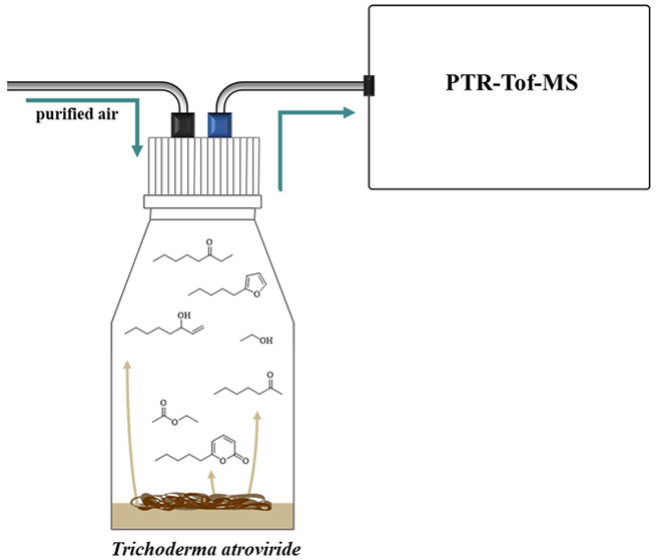

The present study aims to explore the potential application of proton transfer reaction time-of-flight mass spectrometry (PTR-ToF-MS) for real-time monitoring of microbial volatile organic compounds (MVOCs). This investigation can be broadly divided into two parts. First, a selection of 14 MVOCs was made based on previous research that characterized the MVOC emissions of *Trichoderma atroviride*, which is a filamentous fungus widely used as a biocontrol agent. The analysis of gas-phase standards using PTR-ToF-MS allowed for the categorization of these 14 MVOCs into two groups: the first group primarily undergoes nondissociative proton transfer, resulting in the formation of protonated parent ions, while the second group mainly undergoes dissociative proton transfer, leading to the formation of fragment ions. In the second part of this investigation, the emission of MVOCs from samples of *T. atroviride* was continuously monitored over a period of five days using PTR-ToF-MS. This also included the first quantitative online analysis of 6-amyl-α-pyrone (6-PP), a key MVOC emitted by *T. atroviride*. The 6-PP emissions of *T. atroviride* cultures were characterized by a gradual increase over the first two days of cultivation, reaching a plateau-like maximum with volume mixing ratios exceeding 600 ppbv on days three and four. This was followed by a marked decrease, where the 6-PP volume mixing ratios plummeted to below 50 ppbv on day five. This observed sudden decrease in 6-PP emissions coincided with the start of sporulation of the *T. atroviride* cultures as well as increasing intensities of product ions associated with 1-octen-3-ol and 3-octanone, whereas both these MVOCs were previously associated with sporulation in *T. atroviride*. The study also presents the observations and discussion of further MVOC emissions from the *T. atroviride* samples and concludes with a critical assessment of the possible applications and limitations of PTR-ToF-MS for the online monitoring of MVOCs from biological samples in real time.

## Introduction

*Trichoderma* is a genus of filamentous fungi that comprises the best-studied fungal mycoparasites characterized by their ability to stimulate plant-growth and induce systemic resistance in plants against biotic and abiotic stresses.^1,2^*Trichoderma* species are successfully used as biological control agents in agriculture worldwide, making up more than 60% of all registered fungal biopesticides.^3^ One well-studied member is *Trichoderma atroviride*, a plant beneficial fungus with high antagonistic activity toward several fungal plant-pathogens.^2^ While the exact mechanism of how *Trichoderma* species induce systemic resistance in plants is not fully understood, there is strong evidence that microbial volatile organic compounds (MVOCs) facilitate the interaction of the fungus with plants.^4^

MVOCs are small carbon-based molecules with low molecular masses (typically below 300 Da) and low boiling points (typically between 50 and 250 °C) that are produced by microbes as side-products in both primary and secondary metabolism.^5,6^ Consequently, they have high vapor pressures and high volatilities at room temperature. There are numerous known MVOCs belonging to a wide range of chemical classes such as alcohols, aldehydes, hydrocarbons, acids, ethers, esters, ketones, and terpenoids, as well as sulfur- and nitrogen containing compounds.

The unsaturated lactone 6-amyl-α-pyrone (6-PP) is a MVOC known to be a key bioactive compound produced by certain *Trichoderma* species such as *T. atroviride*.^7^ 6-PP is involved as a self-signaling compound in developmental processes of the fungus itself but also shows antifungal activity and regulates plant root architecture, inhibiting primary root growth and inducing lateral root formation.^8,9^ Vinale et al. observed that the treatment of tomato and canola seedlings with 6-PP (and harzianolide) had a positive effect on plant growth and also led to a reduction of disease symptoms caused by the plant pathogens *Botrytis cinerea* and *Leptosphaeria maculans*.^10^ Treatment with 6-PP also reduced the severity of downy mildew infections in grapevines, and there were indications that 6-PP induced plant defense mechanisms.^11^ Similar to most other α-pyrones,^12^ 6-PP might be formed via the polyketide synthase pathway using degradation products derived from the β-oxidation of linoleic acid.^13,14^

Linoleic acid is thought to also serve as precursor for the production of two other important MVOCs, namely, 2-pentylfuran and 1-octen-3-ol via lipid peroxidation.^15,16^ 2-Pentylfuran was found to affect plant growth in a similar way as 6-PP.^11^ 1-Octen-3-ol is an important flavor compound in mushrooms and was reported as a regulating and communication agent in *Trichoderma* species, along with other MVOCs such as 3-octanone.^17^

The monitoring of MVOC emissions during fungal cultivation and their correlation with morphological alterations or other metabolic products can yield information about their origin and role. Online monitoring of MVOC emissions from mycoparasitic fungi could also aid the development of novel biopesticides or the optimization of existing ones by determining the conditions at which the fungus operates most efficiently. Analyzing MVOCs in the headspace of fungal cultures in real-time is a challenging task, since a large number of different substances with a wide range of concentrations can be expected to be produced. The analysis of volatile fungal metabolites therefore requires methods that are sensitive, selective, and offer a broad linear dynamic range. The gold standard in this regard is gas chromatography coupled to mass spectrometry (GC-MS).^18,19^ GC-MS analysis of MVOCs requires sample preparation, e.g., via extraction of MVOCs using the use of solvents,^20^ and preconcentration onto adsorbent tubes^21^ or the use of solid-phase microextraction (SPME).^22,23^ All of these methods require multiple steps of sample preparation, with each step bearing a risk of altering the composition of MVOCs or the loss of MVOCs by condensation, reaction, or degradation. Methods that do not require such extensive sample preparation are desirable, as they mitigate the risks described above.

Gas chromatography coupled to ion mobility spectrometry (GC-IMS) provides good sensitivity toward the detection of MVOCs, can be used without the need for extraction steps, and achieves analysis times of 15–20 min (which can be shortened further depending on the target analytes). The latter two constitute distinct advantages over GC-MS, enabling GC-IMS to be successfully employed for the monitoring of previously identified MVOCs, including several alcohols, ketones, esters, and aldehydes.^14,24,25^ However, the method’s linear dynamic range is limited, as ion signals typically become saturated at volume mixing ratios of over 100 parts-per-billion by volume (ppb_V_). Furthermore, the fast isothermal gas chromatographic separation optimized for the volatile compounds investigated in these studies has drawbacks, as it can impede the elution of less volatile compounds with higher boiling points, such as 6-PP. An analytical technique that is able to overcome these problems is proton transfer reaction mass spectrometry (PTR-MS).^26^ PTR-MS is a soft chemical ionization technique that was introduced in the 1990s and quickly became a popular method for the analysis of VOCs, which provides a number of key advantages. One is simplicity, as no extensive sample preparation is required. A second is its ability to detect VOCs in real-time (within ∼100 ms) with a high sensitivity and limits of detection down to the low-ppt_V_ regime. PTR-MS also offers a wide linear dynamic range, enabling the quantification of volume mixing ratios extending from ppt_V_ to few ppm_V_.

Early PTR-MS instruments exclusively used quadrupole mass analyzers, which have well-known limitations and were gradually replaced by PTR-MS instruments utilizing compact time-of-flight mass analyzers (PTR-ToF-MS).^26^ The use of a time-of-flight mass analyzers allows the full mass range to be recorded, eliminating the need to scan or record parts of the mass range. PTR-MS and especially PTR-ToF-MS instruments have been successfully used for a wide range of applications, including environmental analysis,^27^ food science,^28^ health science,^29−31^ and homeland security.^32−35^ PTR-MS instruments were employed for the detection of MVOCs as early as 2004, with studies targeting both fungi^36^ and bacteria.^29,37−40^ Newer generation PTR-ToF-MS instruments were employed in a limited number of more recent studies on MVOC emissions from fungi^41−43^ and microbial habitats such as soils.^44,45^

In this study, the applicability of PTR-ToF-MS to the quantitative and qualitative online analysis of MVOCs was explored further. The main goal was to establish a method for the online quantitative analysis of the *T. atroviride*-derived key bioactive MVOC 6-PP. A secondary goal was to provide qualitative information on other MVOCs emitted by *T. atroviride*. To this end, the 13 most prominent MVOCs emitted by *Trichoderma* species in previous studies (besides 6-PP)^14,18,24,25^ were also considered in this investigation: 2-pentylfuran, 1-octen-3-ol, ethyl acetate, 2-heptanone, 3-octanone, γ-terpinene, α-phellandrene, 2-methyl-1-propanol, 2-pentanone, 3-methyl-1-butanol, 3-methylbutanal, acetone and ethanol.

## Methods

### MVOC Test Gas Preparation

In order to provide confident identification of MVOCs, it is important to determine which product ions are formed in the proton transfer reactions between the primary H_3_O^+^ ions and the analyte molecules in the first tube (reaction region) of the PTR-ToF-MS. To determine the *m*/*z* values of the product ions associated with any given compound, gas standards were prepared. These used the following chemicals purchased from Sigma-Aldrich/Merck KGaA (Darmstadt, Germany) with known purities: 1-octen-3-ol (≥98%), ethyl acetate (99.9%), 2-heptanone (98%), 2-pentylfuran (≥98%), 3-octanone (≥98%), γ-terpinene (≥95%), α-phellandrene (≥75%), 2-methyl-1-propanol (≥99%), 2-pentanone (99.5%), 3-methyl-1-butanol (99%), 3-methylbutanal (96.6%), acetone (99.9%), ethanol (99.8%), and 6-PP (>96%). Details of the compounds’ CAS numbers, molecular weights, and molecular formulas are provided in Table 1.

**Table 1:** Identity and Product Ion Distribution of the 14 MVOCs Considered in This Study

compounds	CAS	exact mass [u]	product ions (*m*/*z* and relative abundance)		
ethanol	64-17-5	46.042 (C_2_H_6_O)	29.039 (C_2_H_5_^+^) 22%	47.050 (C_2_H_7_O^+^) 78%	
acetone	67-64-1	58.042 (C_3_H_6_O)	59.050 (C_3_H_7_O^+^) 100%		
2-methyl-1-propanol	78-83-1	74.107 (C_4_H_10_O)	41.039 (C_3_H_5_^+^) 8%	57.070 (C_4_H_9_^+^) 92%	
2-pentanone	107-87-9	86.073 (C_5_H_10_O)	87.081 (C_5_H_11_O^+^) 100%		
3-methylbutanal	590-86-3	86.073 (C_5_H_10_O)	69.070 (C_5_H_9_^+^) 85%	87.081 (C_5_H_11_O^+^) 15%	
ethyl acetate	141-78-6	88.052 (C_4_H_8_O_2_)	43.018 (C_2_H_3_O^+^) 14%	61.028 (C_2_H_5_O_2_^+^) 75%	89.060 (C_4_H_9_O_2_^+^) 11%
3-methyl-1-butanol	123-51-3	88.089 (C_5_H_12_O)	43.055 (C_3_H_7_^+^) 48%	57.070 (C_4_H_9_^+^) 6%	71.086 (C_5_H_11_^+^) 46%
2-heptanone	110-43-0	114.104 (C_7_H_14_O)	115.112 (C_7_H_15_O^+^) 100%		
1-octen-3-ol	3391-86-4	128.120 (C_8_H_16_O)	69.070 (C_5_H_8_^+^) 36%	111.117 (C_8_H_15_^+^) 55%	129.128 (C_8_H_17_O^+^) 9%
3-octanone	106-68-3	128.120 (C_8_H_16_O)	129.128 (C_8_H_17_O^+^) 100%		
γ-terpinene	99-85-4	136.125 (C_10_H_16_)	81.070 (C_6_H_9_^+^) 34%	93.070 (C_7_H_9_^+^) 5%	137.133 (C_10_H_17_^+^) 61%
α-phellandrene	99-83-2	136.125 (C_10_H_16_)	81.070 (C_6_H_9_^+^) 35%	93.070 (C_7_H_9_^+^) 11%	137.133 (C_10_H_17_^+^) 54%
2-pentylfuran	3777-69-3	138.104 (C_9_H_14_O)	139.112 (C_9_H_15_O^+^) 100%		
6-amyl-α-pyrone (6-PP)	27593-23-3	166.099 (C_10_H_14_O_2_)	167.107 (C_10_H_15_O_2_^+^) 100%		

The gas standards for all MVOCs (except for 6-PP, see below) were prepared using the total evaporation method. For this, glass bulbs with 1 L volume (Sigma-Aldrich, Darmstadt, Germany) were evacuated for 1 h using a vacuum membrane pump in a dry cabinet (Memmert, Schwabach, Germany) held at 65 °C. Subsequently, 0.5–1 μL of a MVOC was injected into the evacuated glass bulb through a septum (Thermogreen, Merck KGaA, Darmstadt, Germany) undergoing total evaporation. The pressure within the glass bulb was then brought up to atmospheric pressure by means of a 3 L PTFE bag (Tedlar, SKC Ltd., Dorset, UK) filled with high-purity nitrogen (99.999%) connected to the glass bulb. From this gas standard, samples were drawn and further diluted with high-purity nitrogen in 200 mL glass syringes (Socorex Isba SA, Ecublens, Switzerland), eventually yielding gas standards containing approximately 100 ppb_v_ of the MVOC of interest.

Gas standards of 6-PP cannot be prepared using the total evaporation method due to its lower volatility compared to the other MVOCs (associated with its high boiling point of 288 °C). Moreover, it readily adsorbs on surfaces such as the walls of the glass bulb and syringe. Therefore, permeation tubes were prepared by adding 1 mL of 6-PP to a 2 mL glass vial (E. Merck, Darmstadt, Germany) and sealing it using two layers of a polydimethylsiloxane membrane with a thickness of 0.4 mm (Shielding Solutions Limited, UK) held in place by a screw cap with an opening of 5.3 mm (E. Merck, Darmstadt, Germany). This permeation vial was placed in a Schott flask with a volume of 100 mL (Duran GmbH, Germany) with a PTFE screw cap (Bohlender GmbH, Grünsfeld, Germany) fitted with 1/8” inlet and outlet ports, through which the flask was supplied with a flow of purified air at a flow rate of approximately 100 mL/min via perfluoroalkoxy (PFA) tubing. The permeation tube was held at a constant temperature of 30 °C inside a dry cabinet. The weight loss of the glass vial caused by the permeation of 6-PP through the polydimethylsiloxane membrane was measured gravimetrically with a high-precision balance (Sartorius, Göttingen, Germany). Gas samples containing between 30 and 430 ppb_V_ of 6-PP were created by adjusting the flow of purified air through the Schott flask containing the permeation tube to values of between 550 and 45 mL/min, respectively. The samples were introduced directly into the heated inlet line of the PTR-ToF-MS, which was held at 80 °C in order to minimize condensation of 6-PP on the inlet walls.

### Cultivation of *T. atroviride* and Culture Conditions

The cultivation of the mycoparasitic fungus *T. atroviride* strain P1 (ATCC 74058) used in this study has been described in detail elsewhere,^25^ hence only a brief overview is given here. A 6 mm diameter agar plug of the actively growing colony margin from the final preculture was placed upside-down at the edge of the bottom of a 150 mL glass flask (Duran GmbH, Mainz, Germany) containing 25 mL of potato dextrose agar (PDA; Becton, Dickinson and Company, Le Pont De Claix, France). The fungal colony was pregrown at 25 °C for 21 h in complete darkness before the flask was placed in an incubator for headspace sampling. The glass bottles were sealed with Teflon screw-caps (Bohlender, Merck, Vienna, Austria) with a gas inlet and outlet and were flushed with synthetic air at a flow of 5 mL/min.

### Headspace Measurements

#### Headspace Sampling

Headspace measurements were performed on a total of four separate *T. atroviride* cultures (biological replicates) and four glass bottles containing the PDA medium only without fungal inoculation as controls. The headspace measurements were performed over the course of two weeks, with two samples of each type (two *T. atroviride* fungal cultures and two PDA-medium-only) analyzed in parallel in a given working week. The glass bottles were placed in an incubator for the duration of the headspace measurements and connected in a gastight way using 1/16” PFA tubing. The flasks were ventilated by streaming purified air through the flasks at 5 mL/min, which was regulated by mass flow controllers (Bronkhorst, Ruurlo, Netherlands). In order to avoid losses of MVOCs on the walls of glass bottles or tubing, the temperature of the incubator (and hence the temperature of the headspace air) was held at 30 °C, while the water bath (and therefore the temperature of the fungal culture) was kept at 23 ± 2 °C according to the requirements of the fungal growth conditions.

The volatiles emitted from the PDA medium only were also investigated, i.e., without fungal inoculation, so that allowances of any background volatile signals could be made. In order to enable an accurate comparison between the fungal and PDA medium samples, the PDA medium from the same batch was used and the exact sampling times were strictly followed.

For the headspace analysis, the cultivation flask was removed from the incubator and set up with its gas inlet connected to high-purity nitrogen via a T-adapter (for pressure compensation), and its gas outlet connected directly to the PTR-ToF-MS inlet line.

#### PTR-ToF MS Measurements

The underlying principles and methodology of PTR-ToF-MS is described in detail in the literature.^26^ The PTR-ToF-MS instrument used in this study is a state-of-the-art PTR-TOF 6000 X2 (Ionicon Analytik GmbH, Innsbruck, Austria). Its mass resolving power *m*/Δ*m* was ∼6000 for most of the relevant product ions and higher than 5000 for all investigated product ions, which is sufficient to separate most isobaric mass peaks.

The instrument was operated at a hollow cathode current of 4 mA, a sample inlet flow of 35 mL/min, and a drift tube pressure and temperature of 2.6 mbar and 80 °C, respectively. The drift voltage was set at 561 V, resulting in a reduced electric field strength *E/N* (ratio of the electric field strength, *E*, to the total number density, *N*) of 120 townsend (Td) (1 Td = 1 × 10^–17^ V·cm^2^). These settings provide a good balance of sensitivity and avoiding the clustering of primary and analyte ions with molecules such as H_2_O. Furthermore, the settings are similar enough to those used in a large number of previous studies to ensure good comparability with those obtained results. Headspace samples were analyzed either once or twice a day at 22, 43, 47, 66, 71, 90, 94, and 114 h after inoculation. Mass spectral data were recorded for 300 s for each individual headspace measurement, with background signals recorded for 80 s prior to every sample measurement by introducing the high-purity nitrogen supply directly to the PTR-ToF-MS.

#### PTR-ToF MS Data Analysis

Both the gas standard and headspace measurements were processed using the PTR-MS Viewer software (version 3.4.3.12, Ionicon Analytik GmbH, Innsbruck, Austria). The relevant mass spectral peaks of reagent and product ions were fitted using pseudo-Voigt profiles from which the peaks’ positions (used for the identification of MVOCs) and their areas (used as a measure of MVOC abundance) were obtained. A two-point mass axis calibration was performed using the primary regent ion signal at *m*/*z* 21.023 (H_3_^18^O^+^) and the *m*/*z* 330.848 (C_6_H_5_I_2_^+^) signal produced by the internal diiodobenzene standard of the PTR-ToF-MS. All product ion signals of interest were normalized to a primary ion intensity of 10^6^ H_3_O^+^ ions per second using the corresponding H_3_^18^O^+^ signal at *m*/*z* 21.023 in order to account for variations in the primary ion signal between different measurements and to make the reported results comparable to other studies using PTR- MS instruments. The average signal intensity and standard deviation of the normalized ion intensities was calculated using 30 sample points where the ion signal showed little variability (typically toward the end of a sampling period). For the quantification of 6-PP via the *m*/*z* 167.107 (C_10_H_15_O_2_^+^) signal, a background correction was performed by subtracting the background signal of this ion from the calibration and *T. atroviride* culture measurements.

#### Quantitative Analysis of 6-PP

In order to provide a quantitative analysis of 6-PP in the headspace samples, gas standards containing eight different volume mixing ratios were prepared and measured in triplicate together with a background (i.e., 0 ppb_V_ of 6-PP) measurement. After adjusting the flow rate of synthetic air/high-purity nitrogen (see MVOC Test Gas Preparation), at least 20 min was given to ensure a steady state was reached before any measurements were made. This was checked by repeated measurements of the protonated 6-PP parent ion at *m*/*z* 167.107. A linear calibration curve with an ^R2^ of 0.997 was obtained over a range of volume mixing ratios between 0 and 430 ppbv (see Figure S1).

## Results and Discussion

### Product Ions of the MVOCs

The results from the individual gas standard measurements are summarized in Table 1. For each compound, all associated product ions with a minimum abundance of 5% (relative to the most prominent product ion for a given substance) were identified.

The studied MVOCs can be broadly categorized into two groups. The first is those that mainly undergo nondissociative proton transfer and therefore the protonated parent ion is the dominant, and in some cases only, product ion species formed. Compounds that belong in this category are ethanol; the ketones acetone, 2-pentanone, 2-heptanone, and 3-octanone; γ-terpinene; α-phellandrene 2-pentylfuran; and 6-PP. Some members of this group, namely, acetone, 2-pentylfuran, and 6-PP can be expected to be identified easily and with high confidence in biological samples, as there are few other MVOCs that are either isomers or could produce the same product ion species. The same cannot be said for compounds such as ethanol, 2-pentanone, 2-heptanone, and 3-octanone, where ionic interferences are much more likely and in two cases already evident within our selection of MVOCs, namely, *m*/*z* 87.081 (C_5_H_11_O^+^) being produced by both 2-pentanone (100%) and 3-methylbutanal (15%) and *m*/*z* 129.128 (C_8_H_17_O^+^) being produced by both 3-octanone (100%) and 1-octen-3-ol (9%). In the case of the two monoterpenes considered in this study, γ-terpinene and α-phellandrene, two major product ions are generated, namely, the protonated parent ion at *m*/*z* 137.133 (C_10_H_17_^+^, 61% and 54%, respectively) and a fragment at *m*/*z* 81.070 (C_6_H_9_^+^, 34% and 35%, respectively). This fragmentation pattern is typical for monoterpenes, which makes it easy to identify monoterpenes as a compound class. In the present experiment, γ-terpinene and α-phellandrene produced slightly different product ion distributions, so an unknown mixture of both MVOCs could be analyzed by mapping the expected product ion intensities on the observed one. Unfortunately, this is a theoretical scenario with little practical relevance, as monoterpenes are a huge family of structural isomers that produce very similar fragmentation patterns,^46^ and many monoterpenes can conceivably be produced and emitted by microbial organisms.^4,47^ This makes it essentially impossible to identify specific monoterpenes in a biological sample without chromatographic separation or similar analytical techniques with high specificity toward monoterpene species.

The second category is made up by those MVOCs that mainly or exclusively undergo dissociative proton transfer, meaning that the protonated parent ion is a minor product or is not formed at all. This is the case for the branched alcohols 2-methyl-1-propanol, 3-methyl-1-butanol, and 1-octen-3-ol as well as the branched aldehydes 3-methylbutanal and ethyl acetate. The identification of these substances is inherently more difficult, as there are more product ion channels to consider and the fragments are often unspecific organic fragments, which can be produced by a large number of MVOCs or other substances. Besides the examples discussed above, more cases of ionic interferences can be found within the set of MVOCs considered in this study: *m*/*z* 43.018 (C_2_H_3_O^+^), which was produced by both ethyl acetate (14%) and 3-methyl-1-butanol (48%); *m*/*z* 57.070 (C_4_H_9_^+^), which was produced by 2-methyl-1-propanol (92%) and 3-methyl-1-butanol (6%); and *m*/*z* 69.070 (C_5_H_9_^+^), which was produced by 3-methylbutanal (85%) and 1-octen-3-ol (36%).

### MVOC Emissions from *T. atroviride* Samples

The types and quantities of MVOCs produced and released by fungi are dependent not only on essential processes such as the respective growth phase and sporulation but also on environmental factors, such as exposure to light and humidity.^24^ Thus, the measured MVOC signature can change drastically over the course of cultivation. In the following, the findings from the gas standard measurements discussed above will be used to identify and monitor the MVOC emissions from samples of *T. atroviride* cultures. The contribution of the PDA medium to the MVOC ion signals was found to be negligible in all cases except for acetone, which will be discussed separately below. It should be noted that the assignment of MVOCs purely based on the *m*/*z* values and relative intensities of product ions is always ambiguous to a degree; therefore, all assignments made below should be viewed as tentative. As outlined above, the assignment of MVOCs that predominantly undergo nondissociative proton transfer, for example, 6-PP and 2-pentylfuran, can be made with high confidence. This is in contrast to species that predominantly undergo dissociative proton transfer, for example, 2-methyl-1-propanol and 3-methyl-1-butanol, where a lower-confidence identification has to be made.

#### Quantification of 6-PP Production in *T. atroviride*

**Figure 1:**
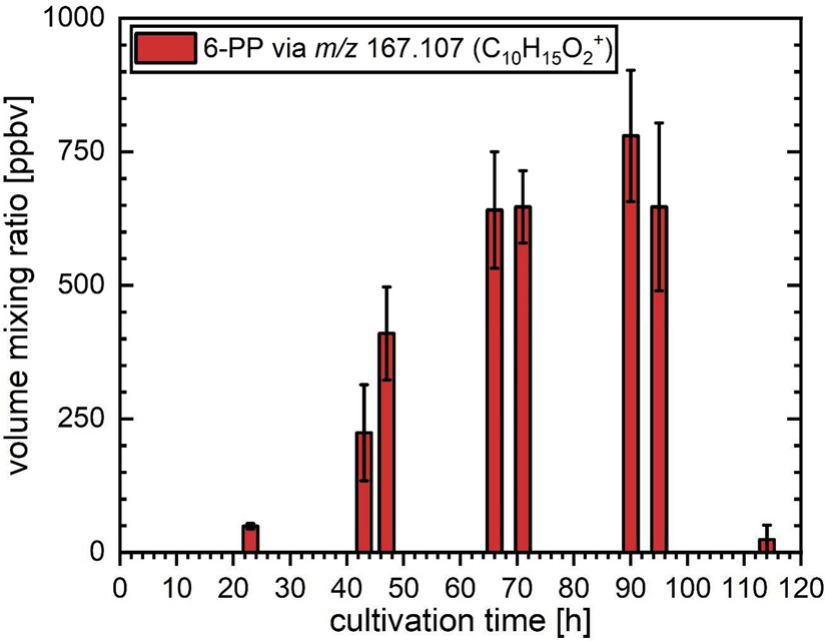
Changes in the intensities of the 6-PP emissions at set cultivation times arising from cultures of *T. atroviride* during five days of measurements. The bar graphs and error bars indicate the average values and standard deviations, respectively, as determined using four independent biological replicates.

Figure 1 displays the volume mixing ratios of 6-PP as determined from the headspace samples taken from the cultivation flasks over five days of incubation and using the calibration described above. Values arising from the four biological replicates are in good agreement, indicated by the relatively low standard deviations (error bars). 6-PP production increased strongly during days one and two between 23 and 47 h after inoculation, when colonization was highly active. The volume mixing ratios reached a broad, plateau-like maximum on days three and four between 66 and 95 h, with the highest values recorded on day four at 90 h after inoculation. Between the fourth and fifth day, a sudden decrease in 6-PP production was observed. The reason for this abrupt decline may lie in the start of sporulation of the fungus. A similar effect was observed by Stoppacher et al.^18^

#### 2-Pentylfuran

**Figure 2:**
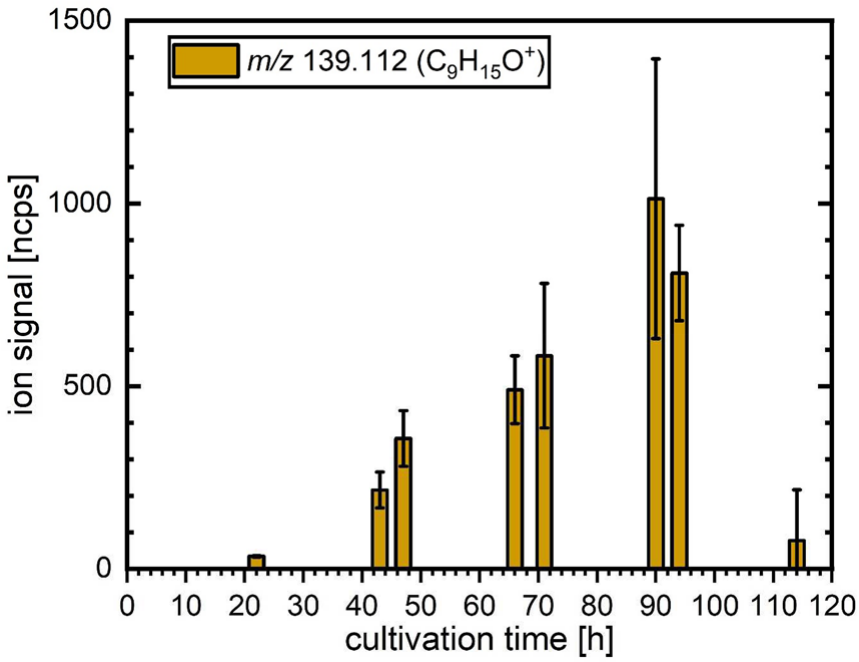
Changes in the ion signal intensities of the product ion at *m*/*z* 139.112 (C_9_H_15_O^+^), assigned to be a result of 2-pentylfuran emission from cultures of *T. atroviride* during the five-day cultivation period. The bar graph and error bars indicate the average value and standard deviation between four independent biological replicates, respectively.

The change in the *m*/*z* 139.112 (C_9_H_15_O^+^) ion intensity assigned to 2-pentylfuran (Figure 2) displayed a temporal evolution very similar to that of 6-PP emissions (see Figure 1). A marked increase between 23 and 66 h was followed by an abrupt decrease between 95 and 114 h after inoculation. The similar temporal patterns of ions associated with 6-PP and 2-pentylfuran during the cultivation time may hint toward a common precursor for these MVOCs’ biosynthesis.^13,15,16^

#### 1-Octen-3-ol, 3-Methylbutanal, 2-Pentanone, and 3-Octanone

**Figure 3:**
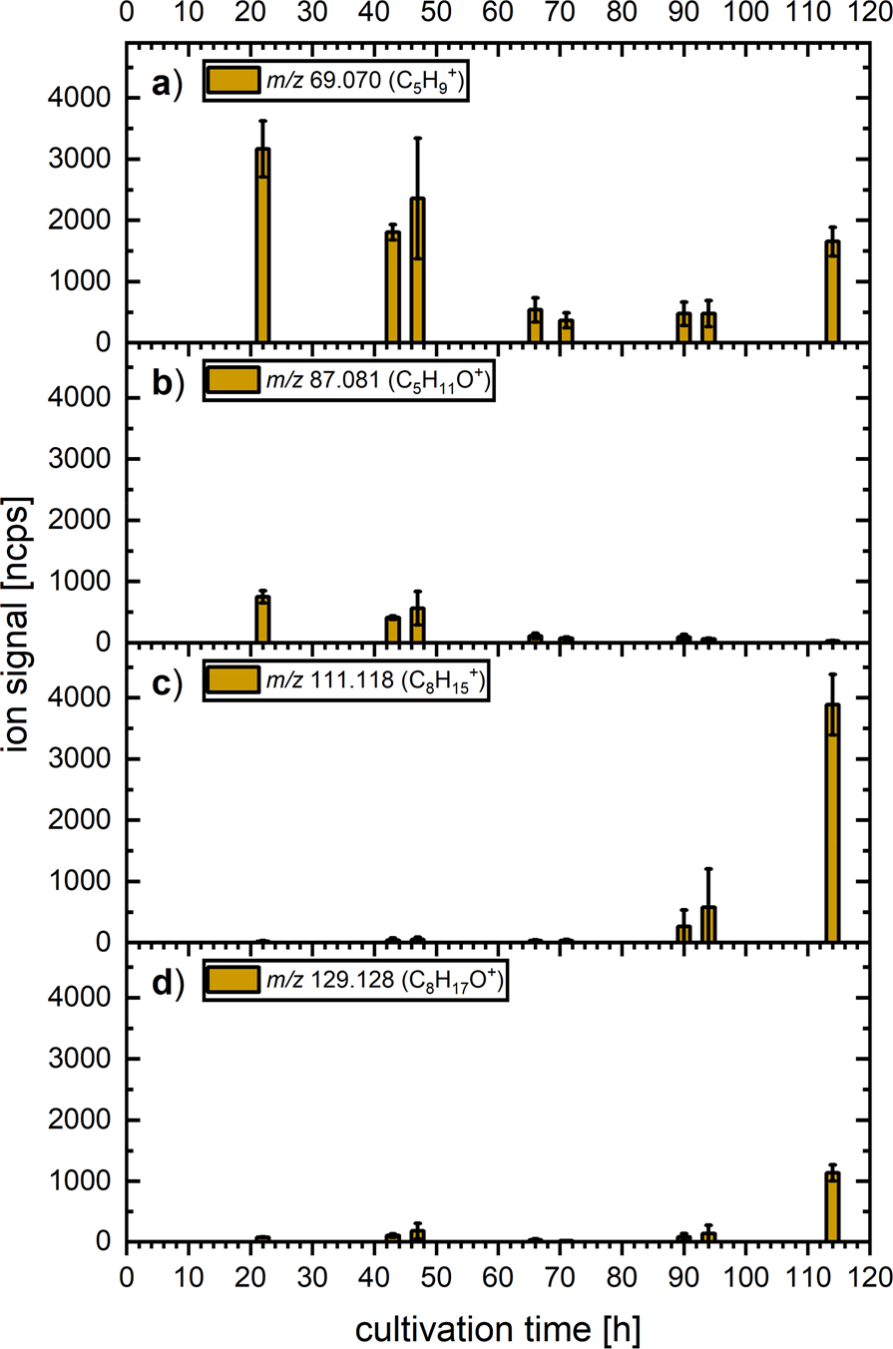
Changes in the product ion signal intensities for *m*/*z* (a) 69.070 (3-methylbutanal and 1-octen-3-ol), (b) 87.081 (2-pentanone and 3-methylbutanal), (c) 111.117 (1-octen-3-ol), and (d) 129.128 (1-octen-3-ol and 3-octanone) arising from cultures of *T. atroviride* during the five-day cultivation period. The bar graph and error bars indicate the average value and standard deviation between four independent biological replicates, respectively.

1-Octen-3-ol, 2-pentanone, 3-methylbutanal, and 3-octanone are important MVOCs previously identified in different studies investigating volatile emissions from *T. atroviride*.^4,24,25^ These compounds produce an overlapping product ion pattern when measured using PTR-ToF-MS, as discussed above (also see Table 1). The changes in the ion intensities of the relevant product ions are displayed in Figure 3.

The signals of *m*/*z* 69.070 (C_5_H_9_^+^) and 87.081 (C_5_H_11_O^+^) (Figures 3a and b) are present at the beginning of the cultivation with relative abundances of 85% and 15%, respectively, which is in perfect agreement with the abundance expected for 3-methylbutanal (see Table 1). This suggests that there are little to no emissions of 1-octen-3-ol or 2-pentanone. However, from the third day onward (66 h), ions at *m*/*z* 69.070 and 87.081 were detected with much reduced intensity compared to days one and two, indicating that the amount of 3-methylbutanal released by the fungi decreased sharply between the second and third day. An increase of the *m*/*z* 69.070 intensity toward the end of the cultivation (without an accompanying increase of *m*/*z* 87.081) likely results from increased 1-octen-3-ol emissions. This is also accompanied by an increase in the *m*/*z* 129.128 (C_8_H_17_O^+^) intensity. This ion is produced by 1-octen-3-ol but is also the only product ion of 3-octanone (Table 1). All three ions associated with 1-octen-3-ol were observed with increasing intensities toward the end of the cultivation period on days four and five, from 90 h until 114 h after inoculation (Figure 3a, c, and d). While the relative intensities between these ions are not in perfect agreement with those in the gas standard measurements, they still agree well qualitatively, indicating an increase in 1-octen-3-ol emission from *T. atroviride* toward the end of the cultivation period. This is consistent with the onset of sporulation between days four and five. The discrepancies in the relative ion yields could be caused by minute amounts of 3-methylbutanal in the case of *m*/*z* 69.070 and 3-octanone in the case of *m*/*z* 129.128. Unidentified compounds not considered in the gas standard measurements, for example, other alcohols or hydrocarbons, could also contribute to these product ion channels.

Unlike alcohols, ketones typically do not fragment upon proton transfer. As previously mentioned, 3-octanone was characterized by a single, but not unique, product ion at *m*/*z* 129.128 (C_8_H_17_O^+^). Unfortunately, the cross-interference of this ion between 1-octen-3-ol and 3-octanone makes it difficult to identify the latter in the fungal samples. The relative intensities of the *m*/*z* 129.128 (corresponding to both 1-octen-3-ol and 3-octanone) and 111.117 (C_8_H_15_^+^) product ion intensities (corresponding only to 1-octen-3-ol) were measured at 30%, 23%, and 29%, respectively, for the last three measurement points (Figure 3c and d). The higher relative abundance of *m*/*z* 129.128 compared to the gas standard measurement (determined to be 17%) suggests that small quantities of 3-octanone were released by the fungal cultures toward the end of the incubation period. The similar trends and increased levels of both 1-octen-3-ol and 3-octanone at the end of the cultivation period exhibit a remarkable alignment with the notion that these two MVOCs are linked to the stress tolerance and conidiation (sporulation) of *T. atroviride*.^17,48^

The identification of 2-pentanone is impeded by an ionic interference caused by its structural isomer 3-methylbutanal. However, the observed relative intensities of the product ions at *m*/*z* 69.07 and 87.081 are consistent with the production of only 3-methylbutanal by *T. atroviride*, as they closely match the product ion relative intensities obtained from the gas standard measurement of this 3-methylbutanal. Thus, the emission of noteworthy quantities of 2-pentanone by the observed *T. atroviride* cultures is deemed unlikely.

#### Monoterpenes

**Figure 4:**
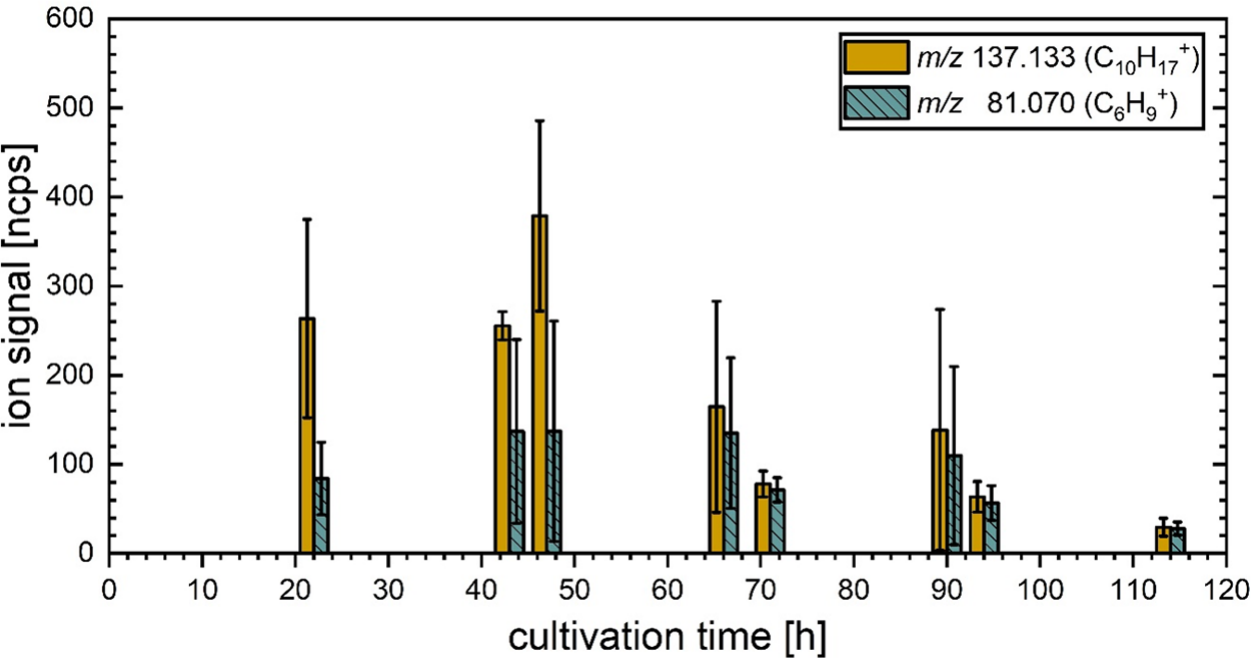
Changes in the product ion signals at *m*/*z* 81.070 and 137.133 arising from cultures of *T. atroviride* at fixed measurement points during the five-day cultivation period. The bar graph and error bars indicate the average value and standard deviation between four independent biological replicates, respectively.

In *Trichoderma*, terpenes function as regulators of growth and stress tolerance.^48^ The two monoterpenes considered in this study, γ-terpinene and α-phellandrene, are both biosynthesized via the intermediate mevalonate pathway starting from acetyl-coenzyme A.^49^ In Figure 4, the changes in the product ion intensities recorded for *m*/*z* 137.133 (C_10_H_17_^+^) and 81.070 (C_6_H_9_^+^) arising from the fungal cultures at the set measuring points are presented. The relatively weak product ion at *m/*z 93.070 (C_7_H_9_^+^) was not evaluated in the biological samples due to interferences from other VOCs overlapping the signal at the same nominal mass, obscuring the *m/*z 93.070 ion signal. However, some interesting observations can still be made: first, the intensities of monoterpene-related product ions are highest on the first two days of cultivation, and the subsequent observed decrease of monoterpene emissions, especially toward the end of the cultivation period, is similar to that observed for 3-methylbutanal. This trend could be connected to fungal sporulation, although the changes were not as drastic as the decline of 6-PP, and, to a lesser extent, 2-pentylfuran or the prominent increase in product ion intensities associated with 1-octen-3-ol. Second, the relative intensities of the *m*/*z* 137.133 and 81.070 product ions are not constant over the course of our observations; in particular, the proportion of *m*/*z* 81.070 is much higher from the third day onward. The change in the relative ion composition, along with the great variability between the biological replicates (indicated by the large standard deviations), suggests that the composition of monoterpenes itself is highly variable, both over the course of the cultivation period and possibly between the individual biological replicates as well. In principle, contributions to the *m*/*z* 81.070 product ion signal from other VOCs, which are not monoterpenes, cannot be ruled out. However, such a contribution is deemed unlikely, as the only known nonmonoterpene VOC to produce *m*/*z* 81.070 ions in reactions with H_3_O^+^ is *cis*-3-hexenal,^50^ but the accompanying characteristic *m*/*z* 99.081 (C_6_H_11_O^+^) ion was not observed at any time in the present study.

### Other MVOCs

#### 2-Heptanone and Ethyl Acetate

**Figure 5:**
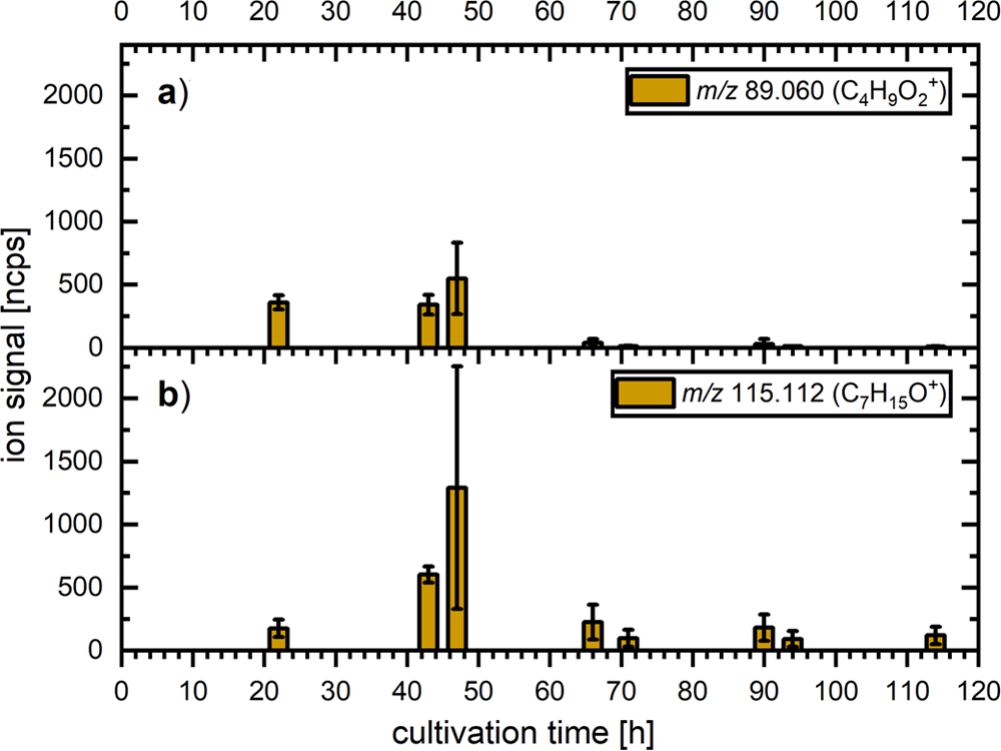
Changes in the product ion intensities at set measuring points for *m*/*z* (a) 89.060 and (b) 115.112 arising from ethyl acetate and 2-heptanone, respectively, which were emitted from cultures of *T. atroviride* during the five-day cultivation period. The bar graph and error bars indicate the average value and standard deviation between four independent biological replicates, respectively.

The changes in ion intensities of *m*/*z* 89.060 (C_4_H_9_O_2_^+^), assumed to originate from ethyl acetate (Figure 5a), and *m*/*z* 115.112 (C_7_H_15_O^+^), assigned to 2-heptanone (Figure 5b), follow a similar trend, with the latter being more pronounced. The intensity of *m*/*z* 115.112 increased during the early cultivation period and peaked at 47 h after inoculation, with values varying greatly between the four biological replicates, as indicated by the large standard deviation. A sudden reduction of the intensity was observed between 47 and 66 h, reaching levels comparable to those observed on day 1. The product ions of *m*/*z* 61.028 (C_2_H_5_O_2_^+^) and 43.018 (C_2_H_3_O^+^) cannot be assigned directly to ethyl acetate in the headspace of fungi samples, since other acetates yield the same product ions as well.^51^

Entering the last three days of cultivation, a marked reduction of the ion signal intensities was detected between 47 and 66 h for both *m*/*z* 115.112 and 89.060, followed by low and relatively stable values during the last three days of cultivation.

#### Acetone

Acetone is a common biomarker that has been previously reported as MVOC emitted from different *Trichoderma* strains,^4,25^ but it is also a commonly used solvent and industrial chemical, as well as a VOC emitted from various natural and anthropogenic sources.^52^ Acetone was detected in both the headspace of fungal cultures and the PDA medium only. Its intensity remained almost constant over the full cultivation period (Figure S2) in the *Trichoderma* cultures. The *m*/*z* 59.050 ion intensity of the PDA-medium-only samples was around 50% that of the fungal culture on the first day, gradually declining to around 10% after five days of inoculation. This indicates that acetone is continuously emitted from the fungal cultures and also, at lower concentrations, from the PDA medium.

#### Other Alcohols

Besides the previously discussed 1-octen-3-ol, the fragmentation patterns of ethanol, 2-methyl-1-propanol, and 3-methyl-1-butanol were investigated, all of which were previously detected in *T. atroviride* cultures.^24,25^ The *m*/*z* 47.050 (C_2_H_7_O^+^) ion, most likely protonated ethanol, showed changes in intensities (Figure S3) similar to those observed for *m*/*z* 89.060 (C_4_H_9_O_2_^+^) and 115.112 (C_7_H_15_O^+^) ions corresponding to ethyl acetate and 2-heptanone, respectively (see Figure 5). The ethanol fragment ion at *m*/*z* 29.039 (C_2_H_5_^+^) could not be evaluated due to interferences with other product ion signals. For the two other alcohols, i.e., 2-methyl-1-propanol and 3-methyl-1-butanol, the product ions of the form C_*x*_H_2*x*+1_^+^ (with 3 ≤ *x* ≤ 5) are inherently unspecific, as they can be produced from a wide variety of alcohols and other MVOCs.^51,53^ In contrast, large ion signals at *m*/*z* 57.070 (C_4_H_9_^+^) and 71.086 (C_5_H_11_^+^), corresponding to 2-methyl-1-propanol and 3-methyl-1-butanol, were detected (Figures S4 and S5), and the relative intensities of the product ions observed from the fungal cultures do not match those determined using the gas standards (Table 1). While the *m*/*z* 41.039 (C_3_H_5_^+^) ion signals are lower than expected from 2-methyl-1-propanol for the majority of data points (Figure S5), the *m*/*z* 43.054 (C_3_H_7_^+^) ion intensity is about twice as high as expected on the first day and about half the expected intensity from 3-methyl-1-butanol on days two through five (Figure S4) in comparison to the gas standard measurements. The analysis of these alcohols highlights the limited analytical capabilities of PTR-ToF-MS when analyzing complex organic mixtures when assignments are solely based on *m*/*z* values.

## Conclusion

In this investigation, the feasibility of using PTR-ToF-MS for the online real-time analysis of MVOCs produced from fungal cultures, without the need for complicated sample preparation, was demonstrated. The product ions generated in the reactions of H_3_O^+^ and 14 MVOCs of interest were determined. The set of MVOCs that was taken into account for this investigation can be classified into two categories. The first category consists of species that primarily undergo nondissociative proton transfer. As a consequence, the main outcome is the formation of predominantly protonated parent ions, with the minimal or nonexistent presence of fragment ions. The second category consists of species that mainly undergo dissociative proton transfer. This process results in the formation of mostly fragment ions, with little or no intact, protonated parent ions. The first group is typically simpler to identify and assign with higher confidence, while the latter are more challenging or, in some cases, impossible to attribute with a reasonable level of certainty due to ionic interferences. Based on these findings, MVOC emissions by cultures of *T. atroviride* were identified and monitored over a cultivation period of five days. Within this investigation, the first quantitative online analysis of the key *T. atroviride* metabolite 6-PP was performed. The volume mixing ratios of 6-PP in the headspace of *T. atroviride* cultures increased from 50 to 400 ppb_v_ during the first two days of cultivation, reaching values between 600 and 800 ppb_v_ on days three and four, which was followed by a marked decrease to about 30 ppb_v_ on day five. The changes in 6-PP emission were found to be highly consistent between the four independent biological replicates. The changes in the intensity of 2-pentylfuran followed that of 6-PP, which is consistent with both MVOCs being derived from a common precursor. The marked decrease of both 6-PP and 2-pentylfuran toward the end of the cultivation period could be connected to the beginning of sporulation of the fungal culture, as it coincided with a marked increase in the product ion intensities corresponding to the emission of 1-octen-3-ol and 3-octanone, two MVOCs associated with conidiation in *Trichoderma* species. 6-PP and 2-pentylfuran could be identified with high confidence in this study and are considered suitable targets for the monitoring of MVOC emissions with PTR-ToF-MS. The identification of MVOCs such as 3-octanone, 1-octen-3-ol and other alcohols highlighted a key limitation of PTR-ToF-MS for the analysis of complex organic mixtures such as MVOCs produced from fungal cultures, namely, identical product ions that result in considerable ambiguity in compound assignment. A more confident identification of MVOCs could be achieved by employing gas chromatographic (GC) pre-separation of compounds, albeit at the cost of real-time analysis. A study exploring the possibilities of combining GC and PTR-ToF-MS will be conducted in the future.

PTR-ToF-MS could be used in agricultural investigations to aid the development of *Trichoderma*-based biopesticides or provide useful information for the optimization of the microbial production of 6-PP in the food industry. The biggest advantage over more established methods such as GC-MS is the ability to track rapid changes in the profiles of MVOCs in real time. This makes it a prime method for investigating processes and mechanisms where such rapid changes might occur, for example, sporulation, changes in carbohydrate or nitrogen metabolism or metabolic switches, such as switching from primary to secondary metabolism. These investigations could also benefit from the possibility of continuous monitoring of biological samples, which PTR-ToF-MS provides. Lastly, the time required for the analysis of a single sample is much reduced using PTR-ToF-MS compared to GC-MS, which greatly enhances the number of biological samples that can be screened in parallel.

## Supporting Information Available

Calibration line used for the quantitative analysis of 6-PP emissions from *T. atroviride* cultures and changes in the product ion intensities (PDF)
