# Supplementary material for: Demonstrating the Applicability of Proton Transfer Reaction Mass Spectrometry to Quantify Volatiles Emitted by the Mycoparasitic Fungus Trichoderma atroviride in Real Time: Monitoring of Trichoderma-Based Biopesticides
Source: J Am Soc Mass Spectrom. 2024 May 6;35(6):1168–77. doi: 10.1021/jasms.3c00456 (PMC11157538; doi:10.1021/jasms.3c00456)
Supplement: Supplementary file 1 — js3c00456_si_001.pdf [file js3c00456_si_001.pdf]

Supporting Information for

Demonstrating the applicability of Proton Transfer Reaction Mass Spectrometry to quantify volatiles emitted by the mycoparasitic fungus *Trichoderma atroviride* in real time: monitoring of *Trichoderma*-based biopesticides

Running title: On-line biopesticide analysis using PTR-MS

Franziska Lochmann[1], Daniel Flatschacher[2], Verena Speckbacher[2], Susanne Zeilinger[2], Valentina Heuschneider[1], Stephanie Bereiter [1], Arne Schiller[1] and Veronika Ruzsanyi[1*]

[1] Institut für Atemgasanalytik, Universität Innsbruck, Innrain 52a and 80-82, A-6020 Innsbruck, Austria

[2] Institut für Mikrobiologie, Universität Innsbruck, Technikerstrasse 25d, A-6020 Innsbruck, Austria

Email of corresponding author: Veronika.Ruzsanyi@uibk.ac.at

Keywords: microbial volatile organic compounds, proton transfer reaction time of flight mass spectrometry, mycoparasitic fungi, *Trichoderma atroviride*, 6-amyl-α-pyrone

Address reprint requests to:

Dr. Veronika Ruzsanyi

University of Innsbruck, Institute for Breath Research

Innrain 52a, 6020 Innsbruck

E-Mail: veronika.ruzsanyi@uibk.ac.at

Telefon: +43 512 507 55603

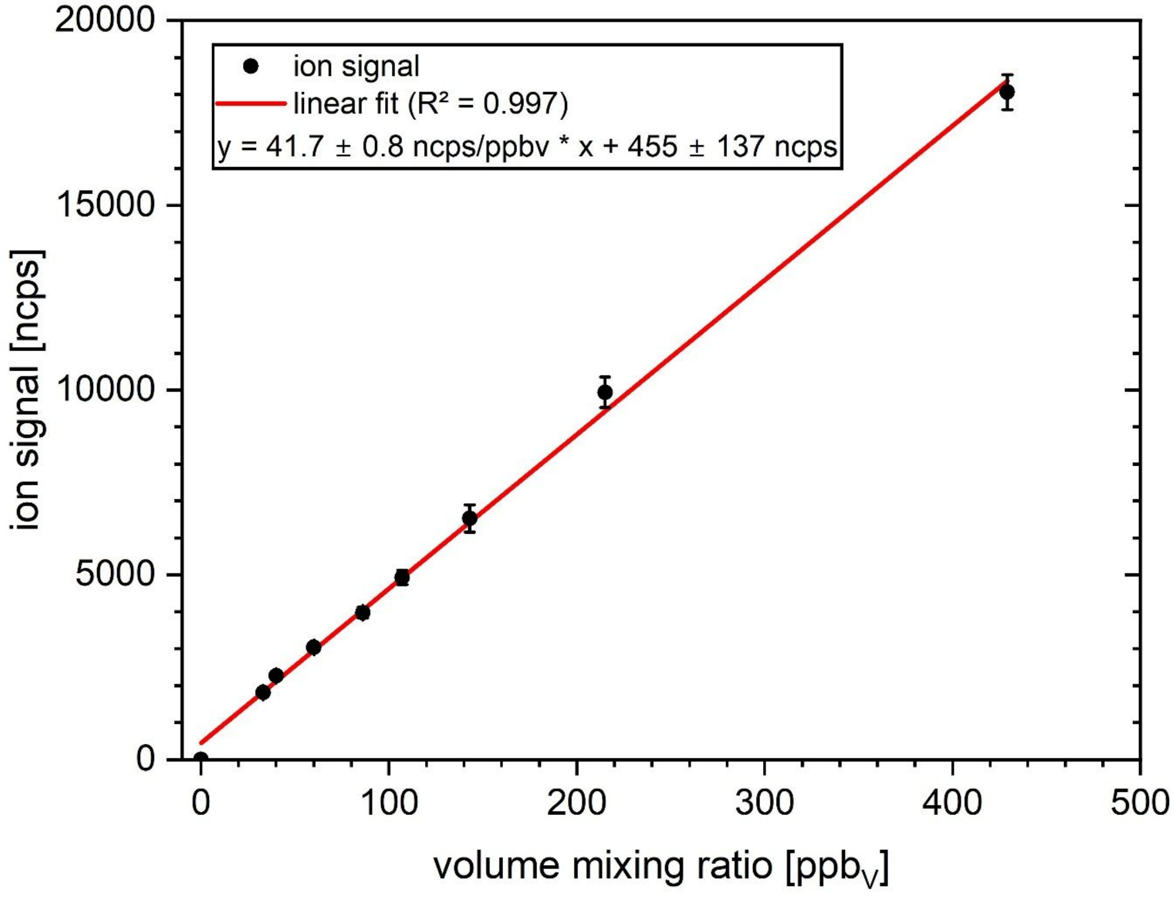

Figure S1: 8-point linear calibration curve used for the quantitative analysis of 6-PP emissions from *T. atroviride* cultures.

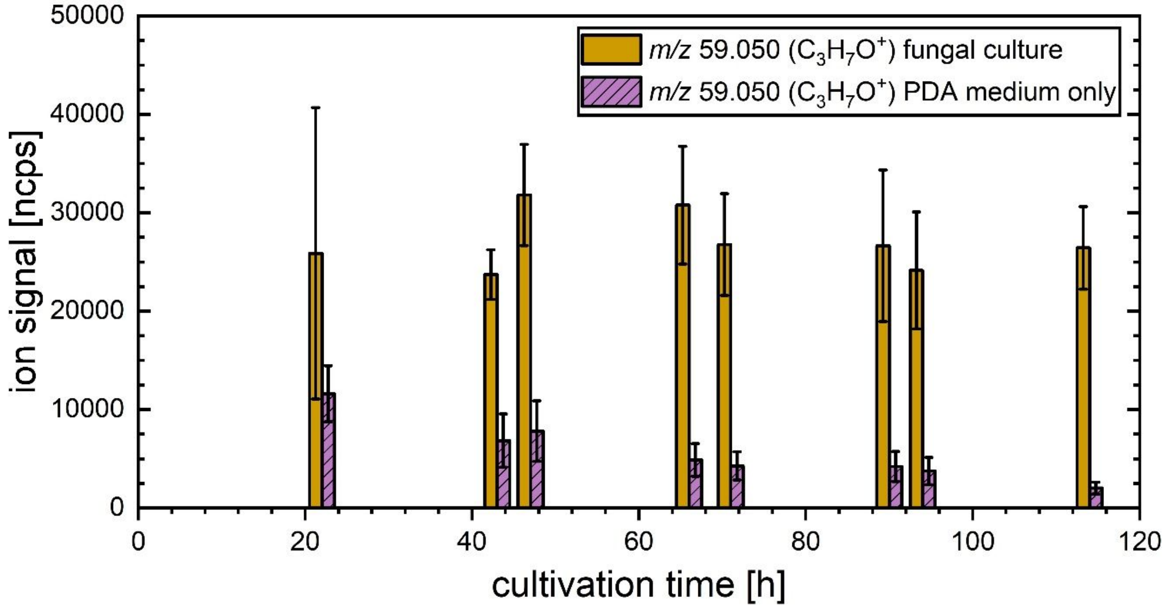

Figure S2: Changes in the product ion intensities at set measuring points for *m/z* 59.050 ion intensity arising from cultures of *T. atroviride* and samples of PDA medium only during the five-day cultivation period. The bar graph and error bars indicate the average value and standard deviation between four independent biological replicates, respectively.

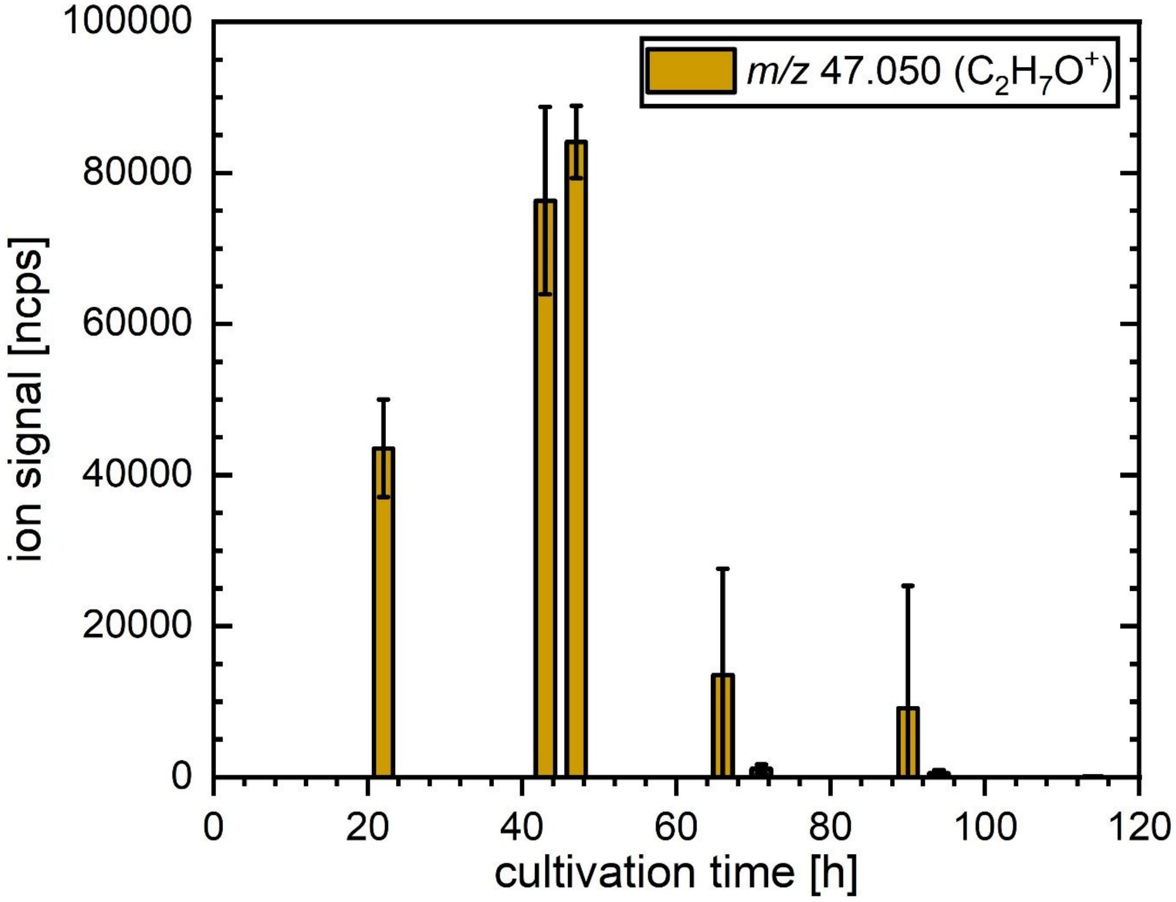

Figure S3: Changes in the product ion intensities at *m/z* 47.050 ion arising from volatile emissions from cultures of *T. atroviride* during the five-day cultivation period. The bar graph and error bars indicate the average value and standard deviation between four independent biological replicates, respectively.

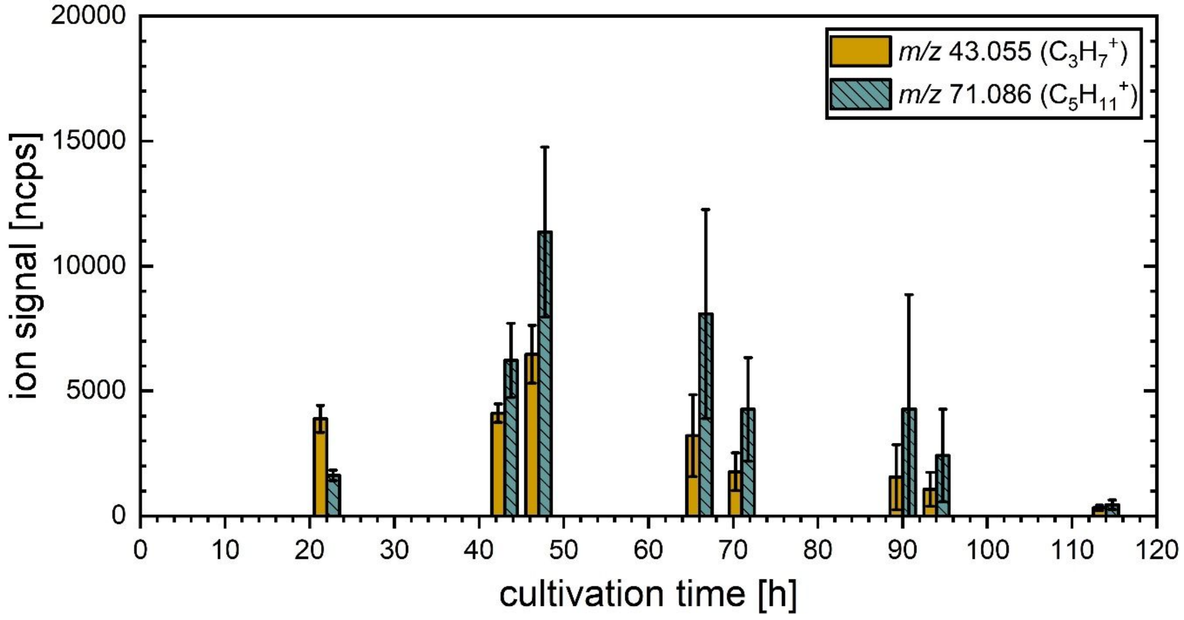

Figure S4: Changes in the product ion intensities at set measuring points for *m/z* 43.055 and 71.086 ion intensities arising from cultures of *T. atroviride* during the five-day cultivation period. The bar graph and error bars indicate the average value and standard deviation between four independent biological replicates, respectively.

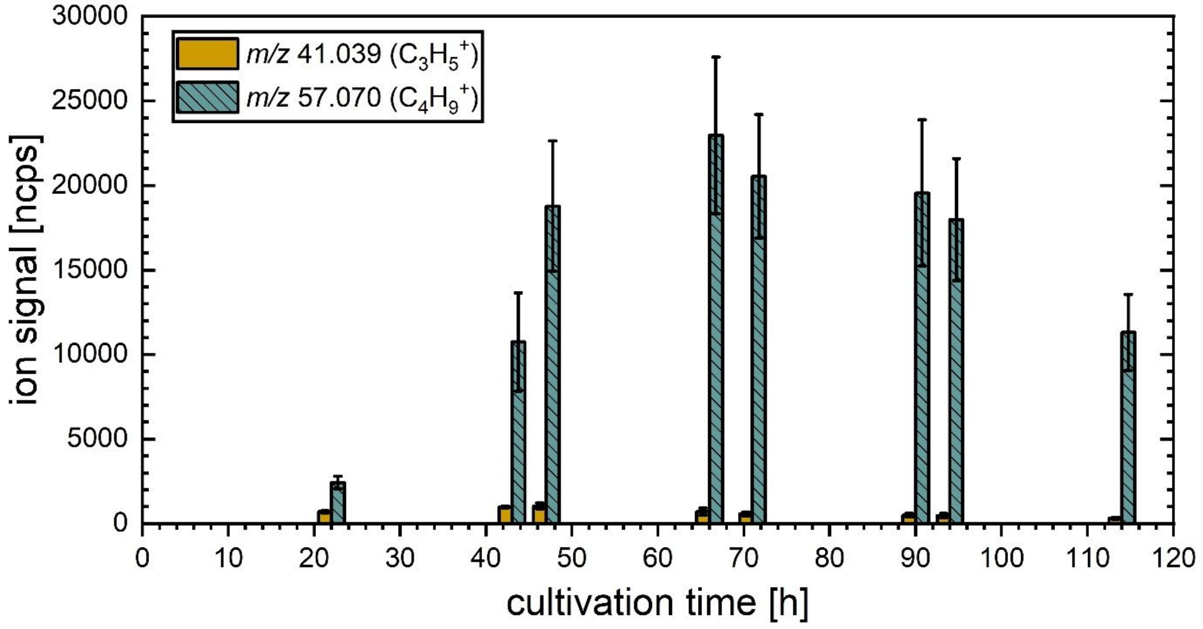

Figure S5: Changes in the product ion intensities at set measuring points for *m/z* 41.039 and 57.070 ion intensities arising from cultures of *T. atroviride* during the five-day cultivation period. The bar graph and error bars indicate the average value and standard deviation between four independent biological replicates, respectively.
